# Vesicle Size Distribution as a Novel Nuclear Forensics Tool

**DOI:** 10.1371/journal.pone.0163516

**Published:** 2016-09-22

**Authors:** Patrick H. Donohue, Antonio Simonetti

**Affiliations:** Civil and Environmental Engineering and Earth Sciences, University of Notre Dame, Notre Dame, Indiana, United States of America; University of Wyoming, UNITED STATES

## Abstract

The first nuclear bomb detonation on Earth involved a plutonium implosion-type device exploded at the Trinity test site (33°40′38.28″N, 106°28′31.44″W), White Sands Proving Grounds, near Alamogordo, New Mexico. Melting and subsequent quenching of the local arkosic sand produced glassy material, designated “Trinitite”. In cross section, Trinitite comprises a thin (1–2 mm), primarily glassy surface above a lower zone (1–2 cm) of mixed melt and mineral fragments from the precursor sand. Multiple hypotheses have been put forward to explain these well-documented but heterogeneous textures. This study reports the first quantitative textural analysis of vesicles in Trinitite to constrain their physical and thermal history. Vesicle morphology and size distributions confirm the upper, glassy surface records a distinct processing history from the lower region, that is useful in determining the original sample surface orientation. Specifically, the glassy layer has lower vesicle density, with larger sizes and more rounded population in cross-section. This vertical stratigraphy is attributed to a two-stage evolution of Trinitite glass from quench cooling of the upper layer followed by prolonged heating of the subsurface. Defining the physical regime of post-melting processes constrains the potential for surface mixing and vesicle formation in a post-detonation environment.

## Introduction

Physical effects of nuclear blasts are recorded in post detonation material. The melting temperatures (ranging between ~700 and ~1200°C) of nearby surficial geologic materials at White Sands were generally exceeded by nuclear blast heat, with some variability with distance from ground zero. The Trinity nuclear test fireball temperature exceeded 8000 K, creating a vapor cloud comprising the bomb, steel tower, and surrounding desert sand [1–3]. Nuclear post-detonation materials, such as Trinitite, are hand-sample scale records of atomic blasts. Relict quartz grains are abundant in Trinitite, although transformation to α-quartz is also observed [4,5]. The brief, 3.1-second high-temperature blast was followed by cooler air brought in by the rising heat, which likely quench-cooled the surface melt [2]. The intact crystal boundaries of *in situ* melted quartz reflects the brevity of the high-temperature conditions [4,6]. However, within this short heating timeframe, the upper centimeter of desert surface was well-mixed with blast material [7], glassy melt pooled and flowed down slopes [1], and long (mm scale) flow bands document mass movement [2,4]. Thus, multiple, complex physical processes were acting in concert to form Trinitite.

**Fig 1:**
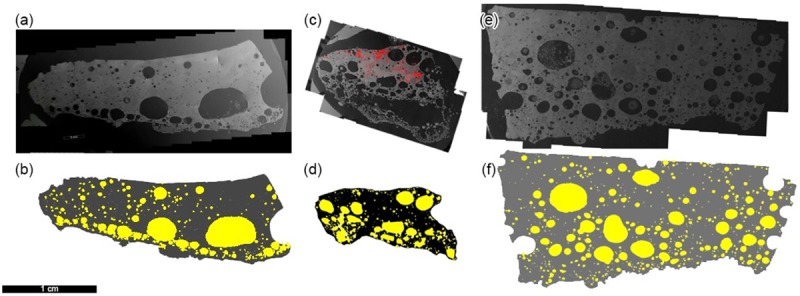
Thin section SEM mosaics and vesicle traces of Trinitite thin sections. (a-b) 4F-5.37; (c-d) 5A-6.06; and (e-f) TS1; All images are oriented with original surface direction at the top. The red region in (c) is the autoradiograph overlay denoting concentrations of radioactive products [10]. This is an indication of “upper” zone of Trinitite, and may be used to determine the original sample surface in poorly-oriented cut samples. The lowest portion of sample 5A-6.06 was not traced due to concerns that the large fragile vesicle was distorted during thin section preparation. Scale is the same for all images (scale bar = 1 cm).

In one scenario of formation, the centimeter-thick Trinitite glass (Fig 1) formed essentially entirely from coalescence of nuclear debris cloud material onto the desert floor [8]. Devolatilization (or volatile escape) of debris cloud material prior to deposition could result in a relatively vesicle-poor melt layer. In an alternate model, the desert surface was melted *in situ*, cooled to a glass by inward-rushing air, and subsequently topped with a small volume of debris cloud material [3,6,9]. The distribution of alpha track activity (linked to Pu and U) and potential anthropogenic contribution (i.e., from the vaporized bomb and tower) is concentrated at the near-surface of Trinitite (Fig 1c), suggesting the vaporized bomb components were primarily introduced during fallout [10]. In some samples, however, the majority of alpha particle activity is concentrated concentrically around the margins of a few mm-sized melt and/or grain fragments. The lack of alpha particle activity within these grains suggests they acquired solely a rim of condensate and did not fully melt and incorporate the blast material. This would appear to support a small volume of fallback contribution to the upper surface of Trinitite. Of course, the distribution of Trinitite is not homogeneous, and neither was the blast fallout—both mechanisms of formation may have occurred at different localities.

The presence of a quench-cooled upper layer, even in a semi-solid state, would trap sub-surface heat and promote melting and volatilization of trace water present in the desert environment [6]. Large ovoid vesicles in Trinitite thin sections appear elongated parallel to the surface (Fig 1), which may result from flattening as a consequence of stagnation during cooling. We hypothesize that the nuclear blast processes resulting in a two-layer Trinitite sample—the upper glassy layer and the lower transitional layer—are reflected in the size distribution and morphology of Trinitite vesicles. To test this hypothesis, we have performed vesicle size distribution calculations on three vertically oriented samples. The Trinitite thin sections investigated are from the most common “green glass” sample morphology. Calculated distances from ground zero, based on the ^152^Eu activity for the three samples, range from ~51 m (4F-5.37 and 5A-6.06) to ~42 m (TS1) [10]. Trinitite thin section mosaics were created from multiple backscattered electron (BSE) images (4F-5.37, 5A-6.06, and TS1) at resolutions of 165–325 pixels mm^-1^ (Fig 1).

## Methods

Trinitite samples were purchased from a commercial provider, the Mineralogical Research Corporation (www.minresco.com), and are maintained at the University of Notre Dame. No permission was required to sample Trinitite at the time of collection (prior to 1953).

Vesicles were traced over thin section mosaics on a separate layer in Adobe Photoshop using a tablet. Partial vesicles intersecting the sample edge (i.e., “open” vesicles) were not included in the analysis or in area calculations. Vesicles were traced from the full area of each sample except for 5A-6.06, for which much of the bottom half of the thin section (below ~8 mm) was too disturbed to accurately trace. Traces were completed based on mosaics of low-magnification (~50× to 100×) BSE images.

Vesicle tracings were exported as monolayer files for analysis in ImageJ, an image-processing program. The length, width, and area of vesicles and the sample were measured using the appropriate image resolution scale. The angle of the long axis and centroid X and Y positions were also measured for subsequent spatial analysis. The raw data for vesicle parameters are provided in the S1 Table. Calculation of a vesicle size distribution requires some assumptions. The original 3D dimensions of vesicles are assumed to be uniform. Length and width of the vesicle set was compared to a series of pre-determined shape files (CSDSlice) to obtain best-fit X-Y-Z scaling dimensions [11]. The possible model shape dimensions range from 1:1:1 (a perfect sphere), 1:1:10 (acicular), to 1:10:10 (tabular). The assumption that the vesicle population is uniform is met if the model of sample data yields a fit value (*R*^*2*^) above 0.8. Stereological corrections to 3D size distributions were performed using the CSDCorrections program [12].

## Results and Discussion

**Fig 2:**
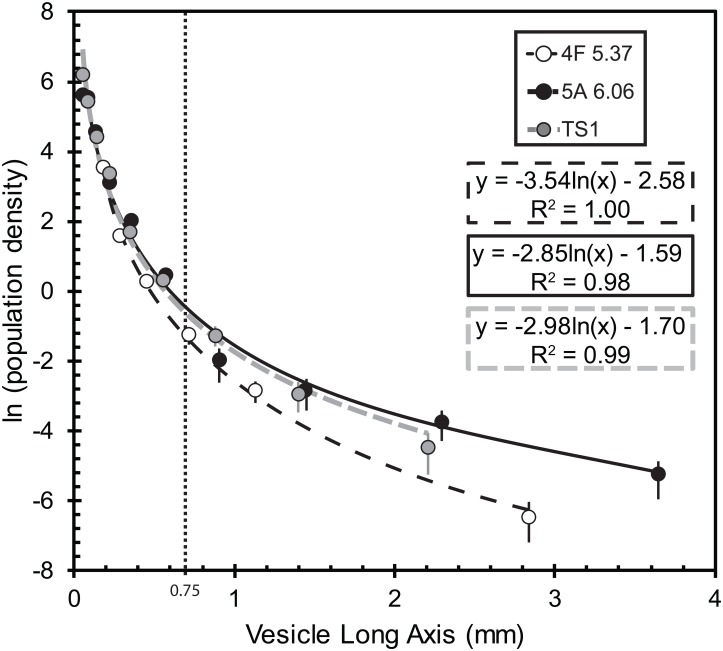
Vesicle population density as a function of size. Best-fit lines demonstrate the logarithmic fit to the data. The cutoff between large and small vesicles is 0.75 mm. By treating the two regions independently, linear fits can be used to calculate slopes and intercepts for the two populations (Table 1). Error bars on points are two sigma, and may be smaller than the symbol size.

Vesicle sizes distributions follow subparallel lognormal distributions between 0.032 and 4.9 mm (Fig 2), and such profile shapes are characteristic of vesicle coalescence. Overall, the upper and lower zones differ mostly in maximum vesicle size and abundance, where the lower zone has 7 to 12% (by volume) more vesicles (Table 1). This difference is a result of a few larger vesicles present in lower zones, as the vesicle distribution profiles are otherwise nearly identical within each sample. One possibility is that prolonged melting in the lower zone increases the probability of vesicle coalescence, and the larger bubbles in the lower zone were protected by incomplete melting, compaction, and mass movement.

**Table 1:** Vesicle size distribution parameters for Trinitite samples.

Sample	distance from GZ*	Sample Area	Vesicle area	n	Max L.	Round-ness	Vol. %	Small vesicles <0.75 mm			Large vesicles >0.75 mm			Shape	shape fit
	*m*	*mm*^*2*^	*mm*^*2*^		*mm*			*slope*	*int*.	*char*. *L*	*slope*	*int*.	*char*. *L*	*x*:*y*:*z*	*R*^*2*^
4F 5.37	51 ± 4	146	33	1179	4.90	0.88	22%	-9.88	5.27	0.10	-3.81	1.49	0.26	1:1:1	0.79
4F 5.37a UZ		117	22	694	4.89	0.88	19%	-16.29	6.33	0.06	-0.79	-3.45	*n*.*d*.	1:1.05:1.05	0.78
4F 5.37a LZ		29	9	501	1.40	0.83	31%	-11.80	7.46	0.08	-3.69	2.82	0.27	1:1.05:1.05	0.85
5A 6.06	51 ± 2	177	58	606	3.71	0.78	33%	-8.71	5.63	0.11	-1.16	-1.06	0.86	1:1.05:1.20	0.91
5A 6.06b UZ		90	24	221	3.10	0.84	26%	-18.38	7.21	0.05	-0.08	-2.30	*n*.*d*.	1:1.1:1.15	0.83
5A 6.06b LZ		87	34	385	3.71	0.74	40%	-9.59	6.00	0.10	-1.59	0.06	0.63	1:1.05:1.25	0.93
TS1	42 ± 1	203	44	710	3.10	0.84	21%	-11.66	6.33	0.09	-2.37	0.66	0.42	1:1:1	0.86
TS1 UZ		122	24	344	3.10	0.86	19%	-10.24	5.81	0.10	-0.68	-1.53	*n*.*d*.	1:1.05:1.05	0.85
TS1 LZ		81	21	365	2.38	0.83	26%	-7.89	5.62	0.13	-2.24	0.68	0.45	1:1:1.1	0.88

*Distances determined by [10].

*UZ* = upper zone; *LZ* = lower zone; *char*. *L* = characteristic length (-1/slope).

*R*^*2*^ is an estimate of goodness of fit to the shape parameters [11].

*n*.*d*.: not determined due to low population yielding poor population statistics.

The distribution of vesicles in Trinitite is generally similar to the random space-filling behavior of vesicles observed in scoria [13]. In contrast, regions with pre-existing crystals should promote heterogeneous vesicle nucleation [13]. Sample 5A-6.06 exhibits the largest difference in spatial distribution between the upper and lower zones. In this sample, the upper zone contains four to five regions (2–4 mm^2^ each) devoid of vesicles, and the calculated nearest neighbor distance is more than double the lower zone (Table 1).

Vesicle sizes and shapes should record details of melt solidification processes, including compaction and fluid flow. Quench cooling is apparent where, in many cases, recently coalesced vesicles retain partial “cusps” at vesicle intersections [14]. Orientation results reveal preferential elongation parallel to the sample surface (±10°). Multiple processes could result in this elongation, including: *1—*Impairment of upward movement by relict grains or density differences between chemical zones; *2—*Trapped bubbles flattened by gravitational compaction; *3—*Pre-existing void spaces preserved by limited melting at increasing depths. Relict grains are common in Trinitite, but most ovoid vesicles are not concentrated nearby. Two of the three samples investigated here do not exhibit significant chemical layering. The ubiquitous elongation in all samples suggests Explanation 2 did contribute to the observed distribution. Explanation 3 could explain some of the heterogeneous distributions of vesicles [6], but likely only the lower zones of Trinitite, where abundances approach typical porosities of loose sand (>30%). Thus, if the lower zones in Trinitite retain partial pore space, additional processes also acted upon the melted sand.

**Fig 3:**
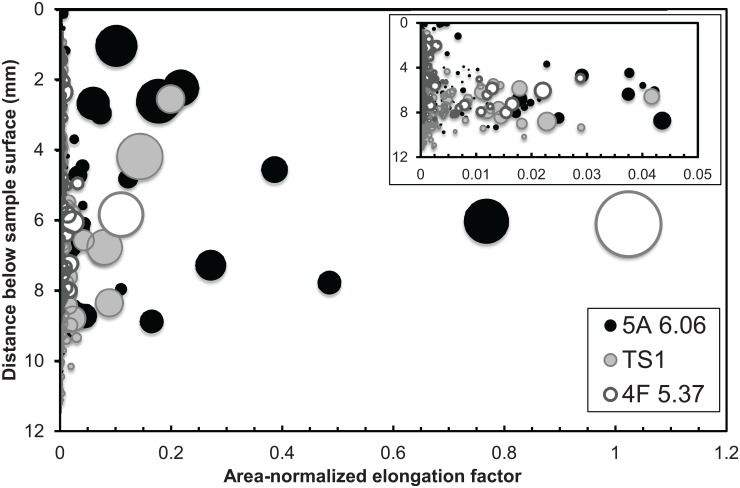
Vesicle location versus deviation from circularity, normalized by area. Large and more ovoid vesicles are more common below ~4 mm (inset region). Symbol size is proportional to vesicle area.

It is shown here that the elongation direction of vesicles is preferentially sub-horizontal throughout Trinitite. Subdividing the sample into upper and lower zones demonstrated the small but distinguishable difference in vesicle size distributions with depth. To further elucidate this, we have calculated the shape factor of each vesicle (0.38–1.34). The deviation from a perfect sphere normalized by area, where shape factor is equal to one, is presented in Fig 3. In this way, small and/or spherical particles approach zero, and large and/or elongated particles are more apparent. Again, we see that a few vesicles describe the difference between upper and lower zones. The majority of large and elongated vesicles are found below ~4 mm depth in Trinitite. However, in this case the distribution is also reflected in smaller vesicles (Fig 3, inset), demonstrating a systematic effect rather than local (sample) heterogeneities. The apparent falling off of vesicles below 6 mm is likely a tracing artifact of less sample area at depth.

**Fig 4:**
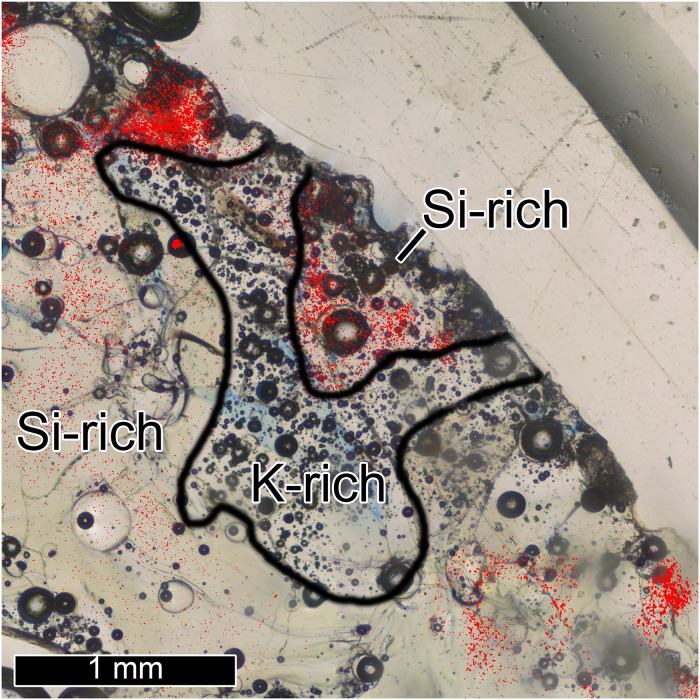
Microscale heterogeneity of components and radiogenic particles in Trinitite. This representative image shows a zone of K-rich melt (bounded by black outline) in sample 5A 6.06 with abundant vesicles and absence of radiogenic particles (red regions indicate high concentrations), surrounded by vesicle-poor Si-rich melt.

Of particular interest, our detailed investigation has noted a tendency for K-rich regions (determined by XRF mapping) to contain significantly higher proportions of vesicles compared with average Trinitite (Fig 4). These regions are also typically isolated and depleted in radiogenic nuclides [10], suggesting they represent incomplete melting of K-feldspar grains. Vesiculated feldspar typically forms in shock stage III impacts, where pressure is ~45–60 GPa [15,16], although lower pressures (< 25 GPa) cause the same effect at higher temperatures [16]. This supports the recent observations of high pressure (~7–8 GPa) in zircon shock features [6,17] and quartz deformation features [17,18].

Our quantitative re-assessment of Trinitite vesicles confirm qualitative observations of distinct textural zones [2,6,7]. The absence of small elongated vesicles in the upper 4 mm of Trinitite suggests a rapid melting and quench cooling event. Larger vesicles may reflect coalescence and pre-existing pore spaces, and so we assume smaller vesicles (up to ~0.75 mm in diameter) are representative of bubble growth. The characteristic vesicle lengths (0.09 to 0.11 mm) for these regions are calculated from the negative inverse of the size versus population density VSD slope [19]. To form this population, estimates of gas diffusion rates from melt suggests vesicle formation continued for at least an hour post-detonation, and was essentially halted within ten hours (Eqns. 14 and 15 of Proust and Fontaine [20]). These estimates use temperature ranges of 725 to 1750°C, and viscosities between 100 and 10000 poise [18], although there are large uncertainties on the viscosities and cooling curves in Trinitite. Heat contribution from radiogenic activity would be required to continue forming vesicles during this time frame. To our knowledge, no previous studies have placed constraints on the post-fallout thermal state for Trinitite.

Trinitite textures reflect their heterogeneous nature of formation in a nuclear blast that incorporated natural material, and a snapshot of this complex history is recorded by vesicles. The thin (<4 mm) vesicle poor surface region of Trinitite, underlain by larger, flattened vesicles, independently supports an origin of combined *in situ* melting and thin fallback layer [9]. Thus we are able to confirm an important constraint on nuclear post-detonation material formation. Vapor cloud fallback, where radiogenic concentrations are assumed to be highest, represents only a fraction of the total volume of Trinitite. Vesiculation likely controls whether fallout remains at the surface or is subsequently mixed.

Naturally, the time scale used to investigate historical PDMs such as Trinitite, does not equate directly to that employed by a nuclear forensic scientist today should a rogue nuclear incident occur. The Trinity blast development was first established through extensive monitoring during the event by a multitude of instrumentation (e.g., video recordings and neutron monitors). Trinitite formation scenarios were later informed by a combination of bulk (*cf*. [21]) and *in situ* analytical methods (*cf*. [2,18]). A real-world scenario would lack many of these observational luxuries, and time or sample volume may be too limited for certain time-intensive analytical methods. As a result, bulk analysis is typically favored, as rapid powdering and dissolution of PDMs can be followed by precise analysis of radioactive components and/or isotopic signatures to identify the bomb source or processing history. However, bulk analysis also removes sample context. In this study, we demonstrated the applicability of an overlooked *in situ* analytical method to the study of Trinitite formation. A growing body of literature has also placed emphasis on using morphological aspects of Trinitite [6,8,10], as well as high resolution in situ geochemical and textural analysis [3,5,6,10,17,18]. Importantly, many in situ investigations require relatively little sample volume; for example, this study required high-resolution photography of a sample thin section or cross section. While the greatest informational yield will come from a combined *in situ* and bulk analytical approach, we emphasize the importance of characterizing sample context prior to destructive analysis.

## Conclusions

Size distributions of small vesicles are a viable method of distinguishing original surface direction in cross section. Several Trinitite samples, including 5A-6.06 and 4F-5.37 investigated here, have regions of high radiogenic activity below the 4 mm upper zone that characterizes fallback [10]. Sample TS1 does not. Blast wave comminution of desert sand is one possible method of mixing bomb material at depth [7]. Alternatively, continued degassing of the molten desert sand would promote near-surface mixing by vesiculation, in addition to increased mobility between phases of different densities [2]. The heterogeneous nature of radiogenic melt distribution suggests both factors contributed to the final characteristics of Trinitite. Yet, the two-stage development process of vesicles is not overprinted by either of these mixing scenarios. Determining the cutoff location for small vesicle formation would serve as a marker for identifying original Trinitite surface orientation.

## Supporting Information

S1 Table Measured vesicle size and shape parameters for Trinitite thin sections. (XLS)
